# Infrared Photodissociation Spectroscopic and Theoretical Study of Mass-Selected Heteronuclear Iron–Rhodium and Iron–Iridium Carbonyl Cluster Cations

**DOI:** 10.3390/molecules30234619

**Published:** 2025-12-01

**Authors:** Jin Hu, Xuefeng Wang

**Affiliations:** Shanghai Key Laboratory of Chemical Assessment and Sustainability, School of Chemical Science and Engineering, Tongji University, 1239 Siping Road, Shanghai 200092, China; hujin328@tongji.edu.cn

**Keywords:** infrared photodissociation spectroscopy, density functional calculation, heterobimetallic carbonyl complex, transition metal–metal bonding

## Abstract

Heterobimetallic iron–group 9 carbonyl cations, FeM(CO)*_n_*^+^ (M = Rh, Ir; *n* = 9–11), were generated in the gas phase via pulsed laser vaporization within a supersonic expansion and characterized by infrared photodissociation spectroscopy in the carbonyl stretching region. By combining experimental spectra with density functional theory simulations, the geometric and electronic structures of these clusters were unambiguously assigned. Mass spectrometry and photodissociation results identified FeM(CO)_9_^+^ as the saturated species for M = Rh and Ir, in contrast to the lighter cobalt analog FeCo(CO)_8_^+^. The FeM(CO)_9_^+^ cations adopt a C_4v_-symmetric singlet ground-state structure with all carbonyl ligands terminally bound, corresponding to a (OC)_5_Fe–M(CO)_4_ configuration. These complexes can be formally described as combination products of the stable neutral Fe(CO)_5_ and cationic M(CO)_4_^+^ fragments. Analyses based on canonical molecular orbitals, Mayer bond orders, and fragment-based correlation diagrams reveal the presence of a dative Fe→M interaction in FeM(CO)_9_^+^, which formally enables the heavier Rh/Ir metal center to attain an 18-electron configuration. However, this bond is weaker than a typical covalent single bond, as the key molecular orbitals involved possess antibonding character. This study provides important insights into the structure and bonding of heteronuclear transition metal carbonyl clusters, highlighting distinctive coordination behavior between late 3*d* and heavier 4*d*/5*d* congeners.

## 1. Introduction

Transition metal (TM) carbonyl complexes have garnered extensive and enduring attention in modern coordination chemistry due to their ubiquitous presence in heterogeneous and homogeneous catalysis, as well as in inorganic and organometallic synthesis [1,2]. Gas-phase TM carbonyls serve as prototypical models for understanding CO chemisorption on metal surfaces, the binding at active sites of catalysts, and the nature of metal–ligand bonding in inorganic and organometallic chemistry [3,4,5,6,7,8,9]. By investigating the structures and chemical bonding of isolated homonuclear or heteronuclear transition metal carbonyl clusters, molecular-level insights into the synergistic effects of high-performance transition metal catalysts in chemical processes are anticipated [7,10,11,12].

A substantial number of studies have been reported on gas-phase homonuclear or heterobimetallic transition metal carbonyl complexes [3,13,14]. For instance, the combination of infrared photodissociation (IRPD) spectroscopy with density functional theory (DFT) calculations has been successfully applied to probe the structures and vibrational frequencies of homoleptic dinuclear and polynuclear TM carbonyl clusters, leading to rapid progress over the past decade [15,16,17,18,19,20,21,22,23]. Studies have shown that for late first-row transition metals, CO tends to bind terminally to the metal, as observed in species such as Fe_2_(CO)_9_^+^, Ni_2_(CO)_8_^+^ and Cu_2_(CO)_6_^+^ [24,25,26]. In contrast, for earlier transition metals, bridging or semi-bridging CO ligands become more prevalent [27,28,29,30]. In particular, for linearly bridging or side-bridging CO ligands, both the carbon and oxygen atoms simultaneously form bonds with metal atoms, acting as four-electron donors. Furthermore, these complexes often adopt asymmetric structures, preferentially satisfying the 18-electron rule for one of the metal centers. Notably, none of these dinuclear metal species appear inclined to form multiple metal–metal bonds. Across all studied systems, only single bonds or half-bonds have been confirmed.

In contrast to the extensive research on homonuclear metal carbonyls, studies on heteronuclear metal carbonyls are considerably scarcer. Photoelectron velocity-map imaging (VMI) has been employed to investigate mass-selected heteronuclear TM carbonyl anions, revealing valuable electronic structure information. Recently, VMI combined with theoretical calculations was utilized to study a series of nickel-containing heterobimetallic carbonyl anions [31,32,33,34]. Infrared photodissociation spectroscopy is compatible with both cationic and anionic transition metal carbonyls. Through IRPD experiments, Zhou et al. determined the structures of CuFe(CO)*_n_*^−^ (*n* = 4–7) [35], finding that these complexes all exhibit a (OC)_4_Fe–Cu(CO)_n−4_ motif, which can essentially be regarded as a formal single bond between an anionic Fe(CO)_4_^−^ fragment and a neutral Cu(CO)_n−4_ fragment. MCu(CO)_7_^+^ (M = Co, Ni) species have also been identified as having staggered (CO)_4,5_M–M′(CO)_3_^+^ structures [36]. The metal–metal interactions involve *σ*-type bonding between (OC)_4_M and M′(CO)_3_^+^. Another iron-containing heterobimetallic carbonyl complex, FeZn(CO)_5_^+^, has been confirmed to adopt a (OC)_5_Fe–Zn configuration with a half-bond formed between Fe and Zn [37]. Studies on FeM(CO)*_n_*^+^ (M = Co, Ni, Cu) revealed that their saturated coordination number is consistently eight, uniformly exhibiting a (OC)_5_Fe–M(CO)_3_^+^ structure [36].

Currently reported heterobimetallic transition metal carbonyls primarily focus on first-row transition metals. To the best of our knowledge, there have been no spectroscopic reports on gas-phase heterobimetallic carbonyl complexes formed between iron and heavier second- or third-row late transition metals. In this work, heteronuclear iron–rhodium and iron–iridium carbonyl cluster cations were generated in a laser ablation source. The cluster ions of interest were mass-selected and investigated via infrared photodissociation spectroscopy. By comparing the experimental spectra with simulated spectra derived from density functional calculations, the cluster structures were assigned, and structural and bonding characteristics were identified.

## 2. Results

**Figure 1:**
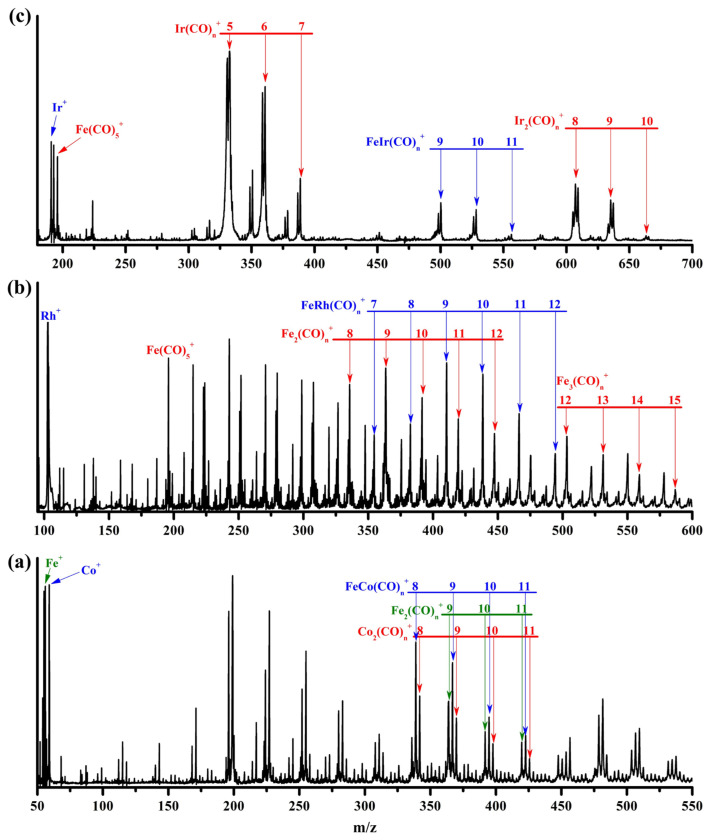
The representative mass spectrum of the cation complexes produced by pulsed laser ablation of an iron–cobalt target (**a**), an iron–rhodium target (**b**), and an iron–iridium target (**c**) in an expansion of 15% CO seeded in helium at a background pressure of 1.4 MPa.

A representative mass spectrum of cations generated by pulsed laser ablation of an iron–rhodium target during the expansion of a CO/He mixture is shown in Figure 1b. The chemical formulas of the various species were assigned based on their mass-to-charge ratios, natural isotopic distributions of the elements, and the most probable carbonyl coordination numbers of the metal centers. The products observed in Figure 1b include homonuclear iron carbonyl complexes Fe*_m_*(CO)*_n_*^+^ (*m* = 1–3), mononuclear rhodium carbonyl complexes Rh(CO)*_n_*^+^, and heterobimetallic iron–rhodium carbonyl complexes FeRh(CO)*_n_*^+^. The species Fe(CO)_5_^+^, Fe_2_(CO)_9_^+^, and Fe_3_(CO)_12_^+^ are well-known saturated coordination compounds, demonstrating superior stability compared to their unsaturated and oversaturated counterparts [24]. In the current mass spectrum, the signal intensities of Fe_2_(CO)_9_^+^ and Fe_3_(CO)_12_^+^ increase dramatically relative to Fe_2_(CO)_8_^+^ and Fe_3_(CO)_11_^+^, respectively, followed by a gradual decrease for Fe_2_(CO)_9–12_^+^ and Fe_3_(CO)_12–15_^+^, indicating that the experimental conditions favor the formation of saturated coordination complexes. Consequently, within the FeRh(CO)*_n_*^+^ series, FeRh(CO)_9_^+^, which exhibits the highest mass abundance, can be inferred to be the saturated species, while the subsequent FeRh(CO)_10,11_^+^ are likely oversaturated weakly bound complexes.

For the iron–iridium target, given the presence of two natural isotopes of iridium, ^191^Ir (37.3%) and ^193^Ir (62.7%), the proportion of iron powder in the mixed target was reduced and experimental parameters were carefully optimized to simplify the mass spectral signals and facilitate unambiguous product identification. This adjustment resulted in a significant suppression of homonuclear iron carbonyl signals and a pronounced emergence of homonuclear iridium carbonyl species Ir*_m_*(CO)*_n_*^+^ (*m* = 1–2), as shown in Figure 1c. The heterobimetallic FeIr(CO)*_n_*^+^ progression was also clearly detected, with FeIr(CO)_9_^+^ exhibiting the strongest mass intensity, suggesting its identity as the saturated coordination species.

It is noteworthy that in previous experiments by Zhou and coworkers, the most intense heterobimetallic carbonyl complex formed between iron and cobalt—the lighter congener of Rh and Ir—was identified as FeCo(CO)_8_^+^, which is saturated but possesses one fewer carbonyl ligand compared to the Rh and Ir analogs [36]. For comparison, metal carbonyl products was prepared under our experimental conditions via laser ablation of an iron–cobalt target (Figure 1a). The results confirm FeCo(CO)_8_^+^ as the most intense signal within the corresponding progression, indicating it is indeed the saturated species, with no evidence for a strongly bound FeCo(CO)_9_^+^ complex. When the nona- and decacarbonyl complexes FeCo(CO)_9,10_^+^ were mass-selected and irradiated under infrared laser in the carbonyl stretching region, significant laser-induced fragmentation occurred (Figure S1). At laser energies of 0.8–1.0 mJ/pulse, the FeCo(CO)_9_^+^ cation underwent rapid dissociation through direct elimination of a CO ligand, resulting in approximately 30% depletion of the parent ions. Under similar irradiation conditions, FeCo(CO)_10_^+^ exhibited direct loss of two CO ligands with similarly high dissociation efficiency, giving rise to an identical infrared photodissociation spectrum to FeCo(CO)_9_^+^. This demonstrates the particularly weak binding of the two outermost CO ligands to the transition metal saturation sphere, thereby supporting the assignment of FeCo(CO)_8_^+^ as the strongly bound complex.

The mass-selected FeRh(CO)_9_^+^ complex exhibited a maximum dissociation efficiency of approximately 5% when irradiated with infrared laser pulses at 1.2 mJ/pulse, which stands in sharp contrast to the behavior observed for FeCo(CO)_9_^+^ (Figure S2a). Such low dissociation efficiency typically characterizes weak multiphoton dissociation processes in strongly bound species, suggesting that FeRh(CO)_9_^+^ possesses a complete coordination sphere. In comparison, the subsequent FeRh(CO)_10_^+^ and FeRh(CO)_11_^+^ complexes underwent rapid elimination of one or two CO ligands, respectively, with dissociation efficiencies approaching 40% (Figure S2b,c). The resulting infrared photodissociation spectra from these processes were identical (Figure S2d), providing direct evidence that FeRh(CO)_9_^+^ is indeed a strongly bound saturated complex. For FeIr(CO)_9_^+^, no dissociation was observed under our laser conditions. Consequently, we investigated FeIr(CO)_10_^+^ and FeIr(CO)_11_^+^, both of which exhibited efficient dissociation patterns similar to those of their rhodium analogs. The identical infrared photodissociation spectra obtained from these species (Figure S3) unambiguously confirm that both are weakly bound solvated complexes with CO messengers. In a nutshell, mass spectrometric and photodissociation spectroscopic observations conclusively demonstrate that the iron-containing heterobimetallic carbonyl complexes with the heavier congeners rhodium and iridium adopt nonacarbonyl species as their saturated structures, while the lighter cobalt analog exhibits an octacarbonyl configuration.

**Figure 2:**
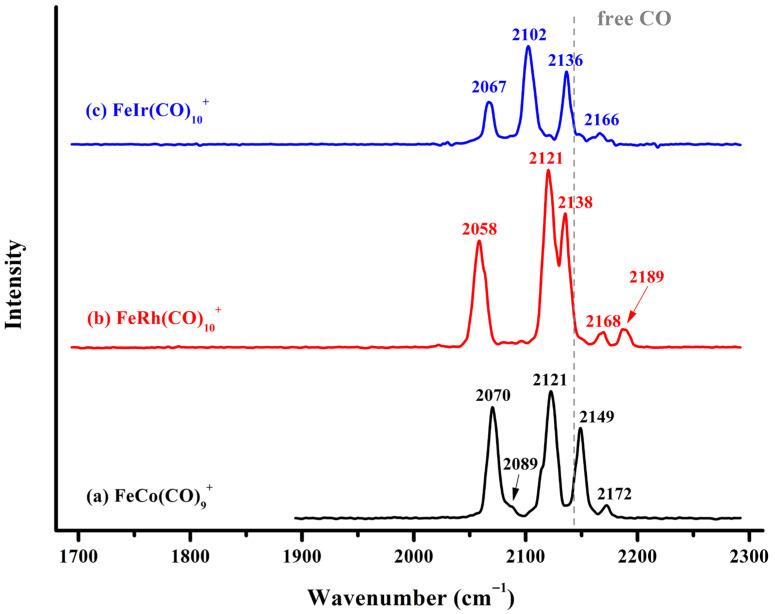
The experimental infrared photodissociation spectra of FeRh(CO)_10_^+^ and FeIr(CO)_10_^+^ via elimination of the outermost physically bound CO in the carbonyl stretching frequency region, leading to the formation of FeM(CO)_9_^+^ (M = Rh, Ir). The vertical dashed line indicates the C–O vibration frequency of free CO at 2143 cm^−1^.

The infrared photodissociation spectra of the FeM(CO)_10_^+^ (M = Rh, Ir) cations and that of FeCo(CO)_9_^+^ are collectively displayed in Figure 2. The infrared spectrum of FeCo(CO)_9_^+^ exhibits five discernible absorption bands at 2070, 2089, 2121, 2149, and 2172 cm^−1^, respectively. The measured spectral structure is highly consistent with that of FeCo(CO)_8_^+^ reported previously [36], although there are slight shifts of 1–5 wavenumbers in the absorption band positions, which may be attributed to the influence of the weakly bound carbonyl tag in the outermost layer. For FeRh(CO)_10_^+^, five well-resolved absorption bands were detected at 2058, 2121, 2138, 2168, and 2189 cm^−1^. Extensive previous infrared photodissociation studies have demonstrated that weakly attached messenger CO ligands typically exhibit a low-intensity band in the 2150–2170 cm^−1^ region [7]. This band appears slightly blue-shifted compared to the characteristic vibration of free CO at 2143 cm^−1^, attributable to the predominantly electrostatic intermolecular interaction between the tag CO ligand and the complex. The remaining characteristic vibrations are attributed to the carbonyl groups of the saturated FeRh(CO)_9_^+^ core. The fact that all these vibrational frequencies lie above 2000 cm^−1^ suggests exclusively terminal bonding modes for all carbonyl ligands. The infrared photodissociation spectrum of FeIr(CO)_10_^+^ exhibits a profile generally similar to that of FeRh(CO)_10_^+^, with four well-resolved absorption bands centered at 2067, 2102, 2136, and 2166 cm^−1^. The absence of any detectable absorption below 2000 cm^−1^ indicates that all carbonyl ligands in FeIr(CO)_10_^+^ are also bound to the metal centers in an end-on manner.

**Figure 3:**
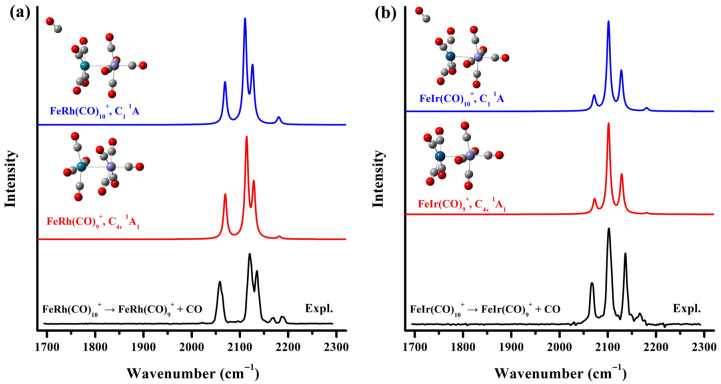
The experimental infrared photodissociation spectra of FeM(CO)_10_^+^ (M = Rh (**a**), Ir (**b**)) and the simulated vibrational spectra of the saturated FeM(CO)_9_^+^ and solvated FeM(CO)_10_^+^ cation complexes in the carbonyl stretching frequency region.

Quantum chemical calculations based on density functional theory were conducted to support the assignments of the vibrational spectra of the observed species and to examine the geometric and electronic structure of the carbonyl complexes. The theoretical investigation commenced with an exhaustive structural search for the target cluster species. Initial structures were constructed by randomly placing carbonyl ligands around the diatomic FeM^+^ core, comprehensively considering carbonyls in various coordination modes, including terminal, bridging, semi-bridging, and side-on bridging configurations, across the three lowest possible spin states of the cluster. Additionally, a series of structures with separated Fe and Rh/Ir atoms were explored. Interestingly, all singlet initial structures, irrespective of bridging carbonyls, converged during optimization to an identical stable configuration, whereas high-spin initial structures failed to converge properly. The resulting stable structure exhibits a (OC)_5_Fe–M(CO)_4_ configuration with a ^1^A_1_ electronic state and C_4v_ symmetry. As shown in Figure 3, the simulated infrared spectrum of this C_4v_-symmetric FeM(CO)_9_^+^ matches perfectly with the experimental photodissociation spectrum of the solvated FeM(CO)_10_^+^ complex, providing unambiguous confirmation of the structures of the observed stable species FeRh(CO)_9_^+^ and FeIr(CO)_9_^+^. To further support these findings, geometry optimization was performed for the solvated FeM(CO)_10_^+^ species. A comparison among the experimental photodissociation spectra of FeM(CO)_10_^+^, the calculated IR spectrum of the saturated FeM(CO)_9_^+^ cations, and that of the CO-tagged FeM(CO)_10_^+^ is presented in Figure 3. The perturbation introduced by the peripheral CO ligand to the geometry and bonding of the fully coordinated FeM(CO)_9_^+^ core is negligible. The geometric structure and simulated spectra of the oversaturated complex FeCo(CO)_9_^+^ have also been optimized and compared with those of FeCo(CO)_8_^+^ (Figure S4). The results similarly indicate that the solvating ligand causes only minimal disturbance to the core framework of the saturated complex. Theoretical and experimental C–O stretching frequencies for the FeM(CO)_10_^+^ (M = Rh, Ir) cations and the FeCo(CO)_9_^+^ complex are summarized in Table 1. The computed values were derived from harmonic frequencies calculated at the B3LYP/def2-TZVPP level, scaled by a factor of 0.968, which was determined from the ratio of the fundamental frequency of free CO (2143 cm^−1^) to its computed value (2214 cm^−1^).

**Figure 4:**
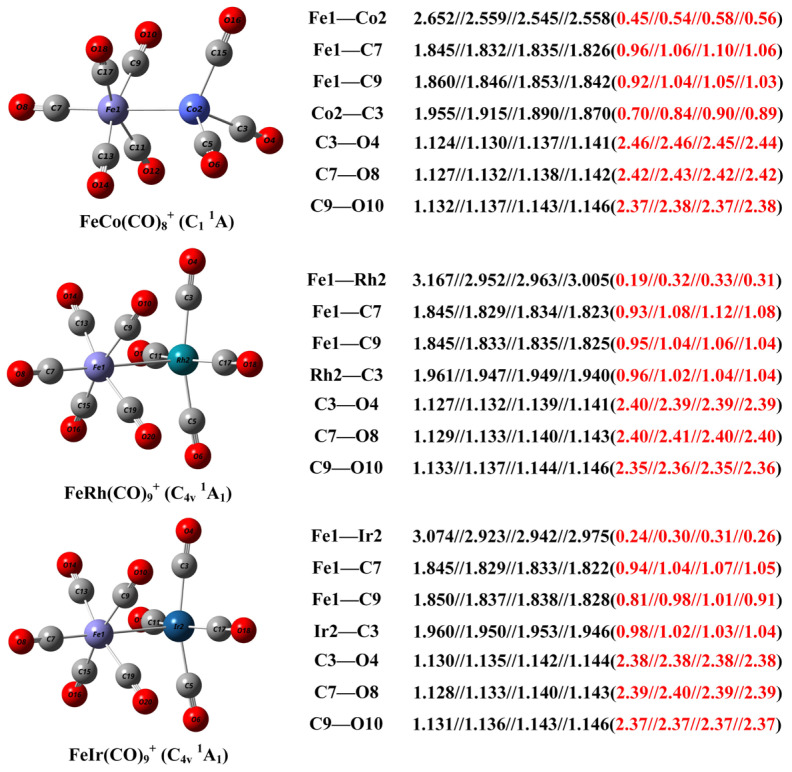
The geometric structure of the FeM(CO)_9_^+^ (M = Rh, Ir) cations and the FeCo(CO)_8_^+^ complex, as well as the calculated bond distance (in Å, black font) and Mayer bond order (red font in parentheses) of selected bonds at the B3LYP//TPSSh//TPSS//PBE level with the def2-TZVPP basis set.

For FeRh(CO)_10_^+^, the absorption band detected at 2058 cm^−1^ comprises two nearly overlapping vibrations of comparable intensity, corresponding to the coupled asymmetric stretching modes of the four carbonyl ligands positioned perpendicular to the Fe–Co bond axis at the iron site (C9–O10; C13–O14; C15–O16; C19–O20, Figure 4). Another coupled asymmetric stretching vibration and the symmetric stretching vibration of these four carbonyls are theoretically predicted at 2161 and 2176 cm^−1^, respectively, yet both exhibit exceedingly weak intensities, rendering them undetectable in the experimental spectrum. Similarly, the intense absorption band at 2121 cm^−1^ is attributed to two of the coupled asymmetric stretching vibrations derived from the four carbonyl ligands bonded to the rhodium center (C3–O4; C5–O6; C11–O12; C17–O18). An additional asymmetric stretching mode is estimated at 2208 cm^−1^, but its low intensity makes it difficult to resolve in the experimental spectrum. The strong band at 2138 cm^−1^ is primarily assigned to the stretching vibration of the carbonyl ligand bound to the iron site along the Fe–Rh bond axis (C7–O8), with minor contributions from coupling effects with the eight approximately perpendicular carbonyl ligands. The weak absorption at 2168 cm^−1^ is attributed to the stretching vibration of the peripherally adsorbed messenger carbonyl ligand, while the most blue-shifted band at 2189 cm^−1^ arises from the symmetric stretching vibration collectively coupled across all nine strongly bound carbonyl ligands. For FeIr(CO)_10_^+^, the vibrational profile is fundamentally consistent with that of FeRh(CO)_10_^+^. The coupled vibrations of the four carbonyl ligands bonded to the iron site and oriented perpendicular to the Fe–Ir bond axis exhibit a slight blue shift compared to those in FeRh(CO)_10_^+^, whereas the vibrational frequency of the carbonyl ligand aligned along the Fe–Ir bond axis remains nearly unaffected. Furthermore, the symmetric stretching vibration collectively coupled across all nine strongly bound carbonyl ligands in FeIr(CO)_10_^+^ exhibits reduced intensity, which consequently prevents its detection in the experimental infrared photodissociation spectrum.

## 3. Discussion

The geometric structure of the FeM(CO)_9_^+^ cations determined through infrared photodissociation spectroscopy experiments combined with theoretical calculations, along with the bond distances and Mayer bond indices of selected bonds, is presented in Figure 4. For comparison, the corresponding structural parameters of the calculated FeCo(CO)_8_^+^ are also provided. A benchmark study of these structural parameters was conducted using B3LYP, TPSSh, TPSS, and PBE as representative functionals for hybrid generalized gradient approximation (GGA), hybrid meta-GGA, meta-GGA, and pure GGA types, respectively. Comparative analysis reveals that B3LYP significantly overestimates bond lengths involving metal atoms compared to the other three functionals, while yielding shorter calculated distances for non-metal C–O bonds. Regarding Mayer bond indices, all four functionals show high consistency for C–O bonds. However, for metal-containing two-center bonds, PBE, TPSS, and TPSSh provide comparable results, whereas B3LYP predictions notably underestimate these values. Previous benchmark studies have similarly demonstrated that while B3LYP performs accurately and with strong generalizability for predicting structures and vibrational frequencies of non-metal compounds [38,39,40], it proves less satisfactory for calculating bond lengths and energies between transition metal atoms [41]. Existing research indicates that the TPSSh functional offers enhanced reliability in handling transition metal–metal and transition metal–nonmetal bonds [42,43]. In the context of infrared photodissociation experiments where carbonyl vibrations are measured, structural assignment through comparison between experimental spectra and simulated spectra calculated at the B3LYP level remains entirely convincing. However, for detailed analysis of structural parameters and chemical bonding, subsequent discussions in this work will utilize computational results obtained at the TPSSh level.

The Fe–Rh bond distance of FeRh(CO)_9_^+^ predicted at the TPSSh level is 2.952 Å, significantly longer than the Fe–Co bond of FeCo(CO)_8_^+^ (2.559 Å). This can be attributed to that, on the one hand, the intrinsic bonding radius of the Rh atom is larger than that of the Co atom, and on the other hand, the Fe–Co bonding interaction here is significantly stronger than the Fe–Rh bonding, as evidenced by the Mayer bond indices of 0.32 for Fe–Rh and 0.54 for Fe–Co. Both the Fe–Ir bond distance and bond order are comparable to those of Fe–Rh, indicating a higher similarity between Rh and Ir compared to the lighter Co congener. The calculated Fe–M bond distances and bond indices suggest significant metal–metal bonding interactions. However, the Fe–Rh and Fe–Ir interactions are relatively weaker compared to Fe–Co, which may be in a certain part related to the number of carbonyl ligands coordinated. The more carbonyl ligands on the Rh and Ir sites enhances interligand electrostatic repulsion. Similarly, in all three complexes, the Fe–C9 bond distance is slightly longer than the Fe–C7 bond, with a smaller Mayer bond order, also due to electrostatic repulsion among the four relatively crowded carbonyl ligands in the plane perpendicular to the Fe–M axis. The Rh–C3 and Ir–C3 bond distances are longer than the Fe–C7 and Fe–C9 bonds, yet they exhibit similar Mayer bond indices, reflecting the significantly larger bonding radii of the heavier Rh and Ir atoms compared to Fe. However, the remarkable similarity in both bond distances and bond indices between Rh–C3 and Ir–C3 suggests significant relativistic contraction in the Ir atom, further indicating that the orbitals involved in carbonyl coordination primarily comprise 6*s* and 6*p* components.

To gain deeper insight into the electronic structures and chemical bonding of the FeM(CO)_9_^+^ cations, the natural population analysis was conducted. As displayed in Table S1, all carbonyl ligands exhibit significant net positive charges, indicating that the interaction with the metal centers is predominated by *σ* donation from carbonyl to metal. The carbonyl aligned with the Fe–M axis (C7–O8) carries a slightly more positive charge compared to those perpendicular to the Fe–M axis on the Fe center, implying stronger *σ* donation and weaker *π* back-donation. This observation is consistent with the vibrational frequency behavior of these two types of carbonyl ligands (Table 1). As later and heavier transition metals compared to Fe, the carbonyl groups bonded to Rh/Ir exhibit more blue-shifted vibrational frequencies relative to those bonded to iron (Table 1), consistent with the formation of so-called non-classical carbonyls where π back-donation is weaker [44,45,46]. This provides the most direct evidence for reduced π back-donation from Rh/Ir to CO compared to Fe. While natural population analysis reveals that the net charge on the Rh/Ir-bound carbonyl (e.g., C3–O4) is more negative than on the Fe-bound carbonyls (Table S1), this apparent anomaly likely stems from the high polarizability of the 4*d*/5*d* metals and the specific partitioning of the highly delocalized electron density in the NPA method. The key factor driving the blue shift remains the weaker π back-donation from the heavier metal centers. From Co over Rh to Ir, the vibrational frequencies of the carbonyl bonded to Fe along the Fe–M axis are 2149, 2138, and 2136 cm^−1^, respectively. This progressively red-shifted trend can also be rationalized by electron transfer from Fe(CO)_5_ to M(CO)_3,4_^+^. According to the calculated Mayer bond orders in Figure 4, the Fe–M bonding weakens gradually from Co over Rh to Ir, indicating reduced electron transfer from Fe to M, which favors greater π backdonation from Fe to CO. Furthermore, neutral iron pentacarbonyl is well established as an 18-electron compound, while Duncan and coworkers have also previously reported the infrared photodissociation spectra of the Rh(CO)*_n_*^+^ cations and determined the Rh(CO)_4_^+^ cation as a stable complex that features a planar square structure and D_4h_ symmetry [46].

**Figure 5:**
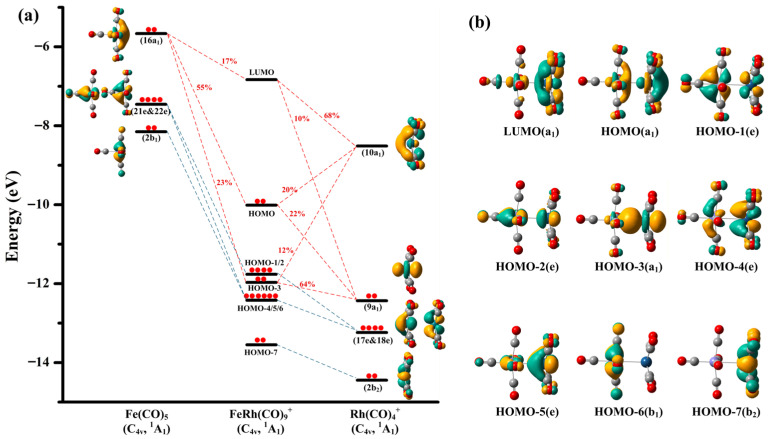
Bonding scheme for the orbital interactions between the neutral Fe(CO)_5_ fragment and the cantionic Rh(CO)_4_^+^ fragment (**a**), as well as the canonical Kohn–Sham molecular orbitals of the C_4v_ structure of these two fragments and the FeRh(CO)_9_^+^ parent complex (**b**) at the TPSSh/def2-TZVPP level. All the molecular orbitals are plotted with isosurfaces = 0.05 a.u. The electronic occupancy (denoted by red solid circles) and orbital symmetry of both the fragments and the parent species are indicated.

The orbital interaction diagram of FeRh(CO)_9_^+^ correlated with the frontier canonical Kohn–Sham valence molecular orbitals is presented in Figure 5. The HOMO-7 of FeRh(CO)_9_^+^, entirely derived from the 2b_2_ orbital of the Rh(CO)_4_^+^ fragment, represents a typical nonbonding orbital. Similarly, the HOMO-6 constitutes a nonbonding orbital originating from the 2b_1_ orbital of the Fe(CO)_5_ fragment. The HOMO-4 and HOMO-5 of FeRh(CO)_9_^+^ form one pair of degenerate orbitals, while the HOMO-1 and HOMO-2 constitute another degenerate pair. These four orbitals arise from the mixing between the degenerate 17e and 18e orbitals of Rh(CO)_4_^+^ and the two e-symmetry degenerate orbitals of Fe(CO)_5_, contributing minimally to inter-fragment bonding due to negligible net electron transfer. Notably, the HOMO-3 of FeRh(CO)_9_^+^ comprises contributions from the 16a_1_ orbital of Fe(CO)_5_ (23%), and the 9a_1_ (64%) and 10a_1_ (12%) orbitals of Rh(CO)_4_^+^. The HOMO orbital is also composed of these three fragment orbitals, with respective contributions of 55% from 16a_1_ of Fe(CO)_5_, 22% from 9a_1_ of Rh(CO)_4_^+^, and 20% from 10a_1_ of Rh(CO)_4_^+^. The LUMO of FeRh(CO)_9_^+^ contains 78% contribution from the Rh(CO)_4_^+^ fragment (9a_1_ accounting for 10% and 10a_1_ for 68%), while the 16a_1_ orbital of Fe(CO)_5_ also contributes 17%. Therefore, the primary orbital interactions between the two fragments can be attributed to the combination of Fe(CO)_5_’s 16a_1_ orbital with Rh(CO)_4_^+^’s 9a_1_ and 10a_1_ orbitals, forming the HOMO-3, HOMO, and LUMO of FeRh(CO)_9_^+^. Note that both the LUMO and HOMO of FeRh(CO)_9_^+^ exhibit antibonding characteristics, explaining why the metal–metal bonding remains weaker than a typical covalent single bond, consistent with the calculated Mayer bond index. For the Fe center, its four *d*-electron pairs, combined with five two-electron donor carbonyl ligands, achieve the favorable 18-electron configuration. The more positive Rh center, with its four *d*-electron pairs and four directly bonded *σ*-donor carbonyls, requires two additional electrons to reach the 18-electron configuration. Consequently, the Fe–Rh interaction in the FeRh(CO)_9_^+^ cation can be described as a dative bonding from Fe(CO)_5_ to Rh(CO)_4_^+^, enabling the Rh center to attain the stable 18-electron configuration. From the perspective of electronegativity, the transfer of electron density from the neutral Fe(CO)_5_ fragment to the cationic Rh(CO)_4_^+^ moiety is fully justified. Given the highly similar vibrational patterns, geometric structures, and bonding parameters, the bonding nature and characteristics of FeIr(CO)_9_^+^ are concluded to be identical to those of FeRh(CO)_9_^+^.

Over the course of this study, another interesting point worth noting is that we observed that when forming homoleptic heterobimetallic carbonyl complexes with Fe, Rh and Ir are nine-coordinate and saturated, in sharp contrast to the eight-coordinate Co. For the lighter Co, the formed eight-coordinate species FeCo(CO)_8_^+^ possesses a triplet electronic state, indicating two unpaired electrons. The iron pentacarbonyl moiety already satisfies the 18-electron configuration, so the unpaired electrons must reside on the Co center, which is supported by the spin population from theoretical calculations (Table S1). The presence of two singly occupied valence orbitals implies that Co can at most achieve a 16-valence-electron configuration. The isolated Co(CO)_3_^+^ fragment has 14 valence electrons. Considering an electron pair provided by the dative bonding from the Fe(CO)_5_ fragment to Co(CO)_3_^+^, the Co center also reaches an “electron-saturated” state. If a nine-coordinate structure, namely (OC)_5_Fe–Co(CO)_4_^+^, were to form, the two singly occupied electrons on Co must pair up and vacate one valence orbital to accommodate an additional carbonyl ligand. The growth of carbonyl complex clusters involves sequential addition of CO ligands; therefore, generating FeCo(CO)_9_^+^ (singlet ground state) from FeCo(CO)_8_^+^ (triplet ground state) requires a spin change, and the kinetic “bottleneck” for this process is not at all unreasonable to expect. In the laser ablation ion source, under rapid cooling via supersonic expansion, FeCo(CO)_8_^+^ may not acquire sufficient energy to overcome the spin crossover barrier and form the singlet product. To further substantiate this kinetic argument, we calculated the binding energy for the addition of CO to triplet FeCo(CO)_8_^+^ to form singlet FeCo(CO)_9_^+^. Our calculations indicate that the singlet state of FeCo(CO)_9_^+^ exhibits C_1_ symmetry, which is a slight distortion of the C_4v_ configuration of FeRh(CO)_9_^+^ and FeIr(CO)_9_^+^. Constraining FeCo(CO)_9_^+^ to C_4v_ geometry would result in a small imaginary frequency. Furthermore, the reaction is found to be exothermic by −0.78 eV at the TPSSh/def2-TZVPP level (Table S2), confirming that the nonacarbonyl is thermodynamically stable. Thus, its absence in the experiment is not a thermodynamic outcome but rather a consequence of a kinetic bottleneck. Similar phenomena have been previously observed in studies of mononuclear vanadium-group and titanium-group carbonyl complexes [47,48,49]. For the heavier Rh and Ir, due to their stronger spin–orbit interactions, spin changes during cluster growth may occur more readily, which explains how these ions form stable nine-coordinate species in our experiments.

## 4. Methods and Materials

Heteronuclear iron–rhodium, iron–iridium, and iron–cobalt carbonyl cluster cations were generated by pulsed laser ablation of composite targets that were prepared by compressing a mixture of iron powder with rhodium, iridium, and cobalt powder (ZhongNuo Advanced Material, Beijing, China) at a molar ratio of 5:1, 2:1, and 1:1, respectively. The molar ratios of the metal mixtures were empirically optimized to enhance the production of the target heterobimetallic carbonyl cations and to simplify the mass spectra. The fundamental 1064 nm output from a Nd:YAG laser (Continuum, Minilite II, Milpitas, CA, USA) was directed onto the rotating metal target via an optical lens, generating a high-temperature metal plasma. Carbonyl complexes were formed through reactions between the plasma and a gas mixture of 15% CO in helium (Qingkuan Chemicals, Shanghai, China), introduced by a pulsed valve (General Valve, Series 9, Parker Hannifin, Cleveland, OH, USA) at a backing pressure of 1.0–1.5 MPa. The resulting species were cooled by supersonic expansion and analyzed using a Wiley–McLaren time-of-flight mass spectrometer. Ions of interest were mass-selected and interacted with tunable infrared radiation from an OPO/OPA system pumped by a Nd:YAG laser (Continuum, Surelite EX). When the IR frequency matched a vibrational mode of the cluster, photon absorption led to dissociation of weakly bound ligands. Since typical bond energies exceed the energy of a single IR photon, the tagging technique with weakly bound messenger molecules was employed to obtain the IR photodissociation spectra indirectly. Thus, solvated weakly bound complexes were mass-isolated for photodissociation studies.

Fragment and parent ions were reaccelerated and analyzed by a second tandem time-of-flight mass spectrometer. IR photodissociation spectra were recorded by monitoring fragment ion yield as a function of IR wavelength, scanned in 2 cm^−1^. steps. The IR pulse energy ranged between 0.6 and 1.2 mJ/pulse.

First principle density functional theory (DFT) calculations were conducted using the Gaussian 09 computational package [50]. The hybrid B3LYP functional [51,52] is the most extensively used density functional for structural optimization and harmonic vibration frequency analysis, which has been demonstrated to be able to provide reliable predictions on the structures and vibrational frequencies of transition metal-containing compounds [38,40,53]. Preliminary structure search for each carbonyl complex was conducted using the def2-SVP basis set at the B3LYP level [54,55], starting from multiple initial configurations with CO ligands randomly arranged around the exposed diatomic FeM^+^ core. Various bridging carbonyl motifs and three lowest spin states were evaluated for each candidate isomer. To improve the accuracy of energy and bond length predictions, the resulting low-lying candidates were further reoptimized using the def2-TZVPP basis set at the PBE [56,57,58], B3LYP, TPSS [59], and TPSSh [60,61] level of theory, respectively. PBE, B3LYP, TPSS, and TPSSh served as representatives of pure generalized gradient approximation (GGA), hybrid GGA, meta-GGA, and hybrid meta-GGA functionals, respectively, to provide a benchmark study. After each optimization, the harmonic vibration frequency was carefully checked to confirm that a true minimum point was obtained, and the zero point vibration energy (ZPVE) was derived. The computational spectra were scaled by a factor of 0.965 and widened by the Gaussian-type curve with a full width at half-maximum (FWHM) of 4 cm^−1^. The chemical bonding properties were analyzed by employing several methods including Mayer bond order (MBO) [62], natural population analysis (NPA) [63], orbital component analysis based on natural atomic orbital (NAO) [64], and orbital correlation based on charge decomposition analysis (CDA) [65]. These analyses were carried out with the Multiwfn software 3.8 package [66,67].

## 5. Conclusions

In summary, heterobimetallic iron-based transition metal carbonyl cations of the form FeM(CO)*_n_*^+^ (M = Rh, Ir; *n* = 9–11) were generated in the gas phase via pulsed laser vaporization within a supersonic expansion and investigated by infrared photodissociation spectroscopy in the carbonyl stretching region. Their geometric and electronic structures were unambiguously determined by comparing the experimental spectra with simulated spectra obtained from density functional theory calculations. Combined laser-induced fragmentation mass spectrometry and infrared photodissociation spectroscopy revealed that the observed FeRh(CO)_9_^+^ and FeIr(CO)_9_^+^ are the saturated complexes, unlike their lighter congener cobalt, for which the octacarbonyl FeCo(CO)_8_^+^ is the saturated species. The dinuclear FeM(CO)_9_^+^ cations adopt a singlet ground-state structure with C_4v_ symmetry, where all carbonyl ligands are bound to the metal centers in a terminal, end-on fashion, collectively forming a (OC)_5_Fe–M(CO)_4_ configuration. Thus, each FeM(CO)_9_^+^ complex can be formally viewed as the product of two stable metal carbonyl fragments: neutral Fe(CO)_5_ and cationic M(CO)_4_^+^. Orbital analysis, Mayer bond orders, and fragment-based correlation diagrams reveal the presence of a dative Fe→M interaction in FeM(CO)_9_^+^, which formally enables the heavier Rh/Ir metal center to attain an 18-electron configuration. However, this bond is weaker than a typical covalent single bond, as the key molecular orbitals involved possess antibonding character. These findings provide important new insights into the structure and bonding of heteronuclear transition metal carbonyl clusters.

## Figures and Tables

**Table 1:** Comparison of the calculated carbonyl stretching frequencies (in cm^−1^) of the FeM(CO)_10_^+^ (M = Rh, Ir) cations and FeCo(CO)_9_^+^ complex at B3LYP/def2-TZVPP level of theory with the experimental values in the present work. IR intensities are listed in parentheses in km/mol, and all frequencies are scaled with a factor of 0.968.

Complex	Expl.	Calc.	Mode
FeRh(CO)_10_^+^	2058	**2068(5** **87)** **, 2068(582) ^a^**	Four carbonyls (at Fe) asym-str.
		2079(0)	Four carbonyls (at Fe) asym-str.
		2093(3)	Four carbonyls (at Fe) sym-str.
	2121	**2114(** **1225)** **, 2114(1330)**	Four carbonyls (at Rh) asym-str.
		2124(33)	Four carbonyls (at Rh) asym-str.
	2138	**2128(** **1378)**	one carbonyl (at Fe) str.
	2168	**2181(** **85)**	one carbonyl (tag) str.
	2189	**2182(** **53)**	Nine carbonyls sym-str.
FeIr(CO)_10_^+^	2067	**2071(** **283)** **, 2072(265)**	Four carbonyls (at Fe) asym-str.
		2085(0)	Four carbonyls (at Fe) asym-str.
		2097(22)	Four carbonyls (at Fe) sym-str.
	2102	**2102(** **1600)** **, 2102(1709)**	Four carbonyls (at Ir) asym-str.
		2114(24)	Four carbonyls (at Ir) asym-str.
	2136	**2128(** **1460)**	one carbonyl (at Fe) str.
	2166	**2180(** **87)**	one carbonyl (tag) str.
		2181(21)	Nine carbonyls sym-str.
FeCo(CO)_9_^+^	2070	**2071(860), 2072(819)**	Four carbonyls (at Fe) asym-str.
		2080(18)	Four carbonyls (at Fe) asym-str.
	2089	**2104(166)**	Four carbonyls (at Fe) sym-str.
	2121	**2125(751), 2127(687)**	Three carbonyls (at Co) asym-str.
	2149	**2138(808)**	one carbonyl (at Fe) str.
		2170(62)	one carbonyl (tag) str.
	2172	**2179(66)**	Eight carbonyls sym-str.

^a^ the modes (bold) were observed.

## Data Availability

Data are contained within the article.

## Supplementary Materials

The following supporting information can be downloaded at: https://www.mdpi.com/article/10.3390/molecules30234619/s1, Figure S1: the laser-induced photofragmentation mass spectra and resulting infrared photodissociation spectra of mass-selected FeCo(CO)_9,10_^+^; Figure S2: the laser-induced photofragmentation mass spectra and resulting infrared photodissociation spectra of mass-selected FeRh(CO)_9–11_^+^; Figure S3: the laser-induced photofragmentation mass spectra and resulting infrared photodissociation spectra of mass-selected FeIr(CO)_10,11_^+^; Figure S4: comparison of the experimental infrared photodissociation spectrum of FeCo(CO)_9_^+^ and the calculated spectra of FeCo(CO)_8,9_^+^; Table S1: the calculated charge population of FeM(CO)_9_^+^ based on natural population analysis; Table S2: calculated thermodynamic energy of free CO, triplet FeCo(CO)_8_^+^, and singlet FeCo(CO)_9_^+^, as well as the relative energy of singlet FeCo(CO)_9_^+^ to FeCo(CO)_8_^+^ plus CO; Table S3: the cartesian coordinates (Å) of the structures of FeM(CO)_9_^+^ optimized at B3LYP/def2-TZVPP level.
