# Supplementary material for: Infrared Photodissociation Spectroscopic and Theoretical Study of Mass-Selected Heteronuclear Iron–Rhodium and Iron–Iridium Carbonyl Cluster Cations
Source: Molecules. 2025 Dec 1;30(23):4619. doi: 10.3390/molecules30234619 (PMC12693129; doi:10.3390/molecules30234619)
Supplement: Supplementary file 1 [file molecules-30-04619-s001.zip › molecules-4007765-supplementary.pdf]

Supplementary Materials

Infrared Photodissociation Spectroscopic and Theoretical Study of Mass-selected Heteronuclear Iron-Rhodium and Iron-Iridium Carbonyl Cluster Cations

Jin Hu , Xuefeng Wang*

Shanghai Key Laboratory of Chemical Assessment and Sustainability, School of Chemical Science and Engineering, Tongji University, 1239 Siping Road, Shanghai 200092, China

* Email: xfwang@tongji.edu.cn

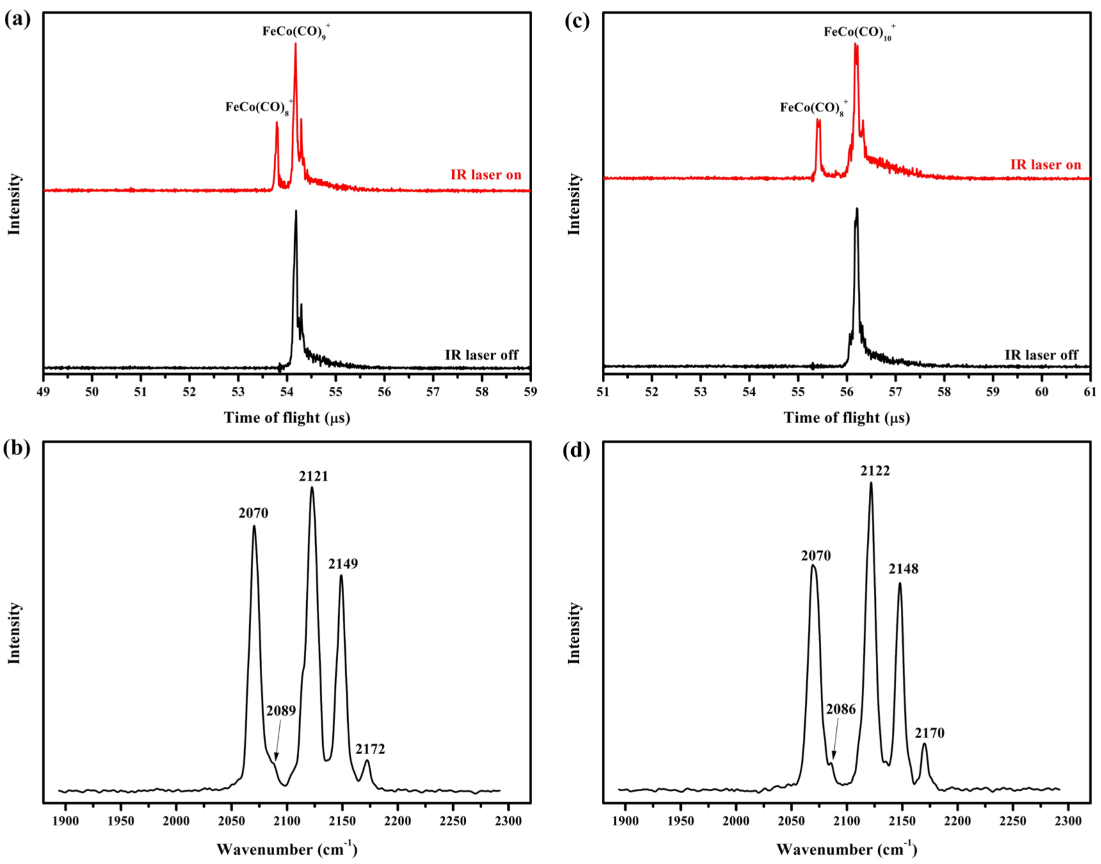

Figure S1. The laser-induced photofragmentation mass spectrum of mass-selected $FeCo(CO)_9^+$ (a) and the resulting infrared photodissociation spectrum (b); the laser-induced photofragmentation mass spectrum of mass-selected $FeCo(CO)_{10}^+$ (c) and the resulting infrared photodissociation spectrum (d).

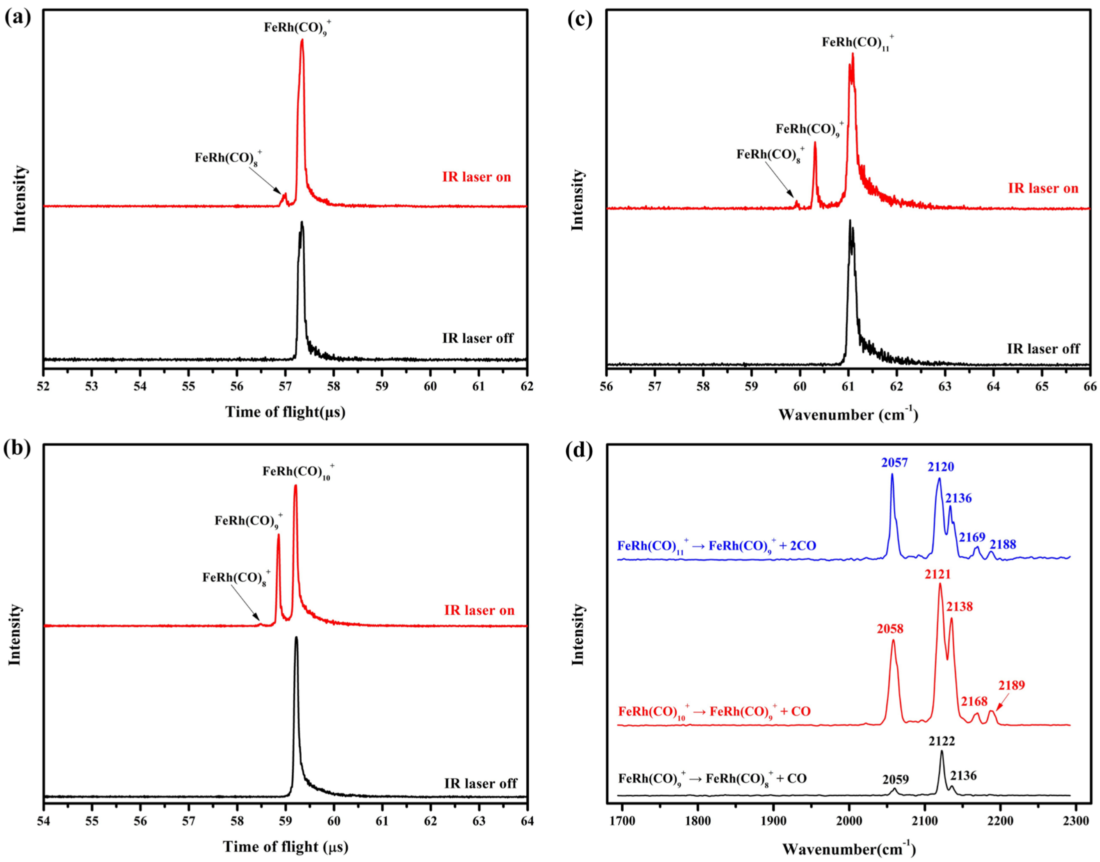

Figure S2. The laser-induced photofragmentation mass spectra of mass-selected $FeRh(CO)_9^+$ (a), $FeRh(CO)_{10}^+$ (b), $FeRh(CO)_{11}^+$ (c), as well as the resulting infrared photodissociation spectra (d).

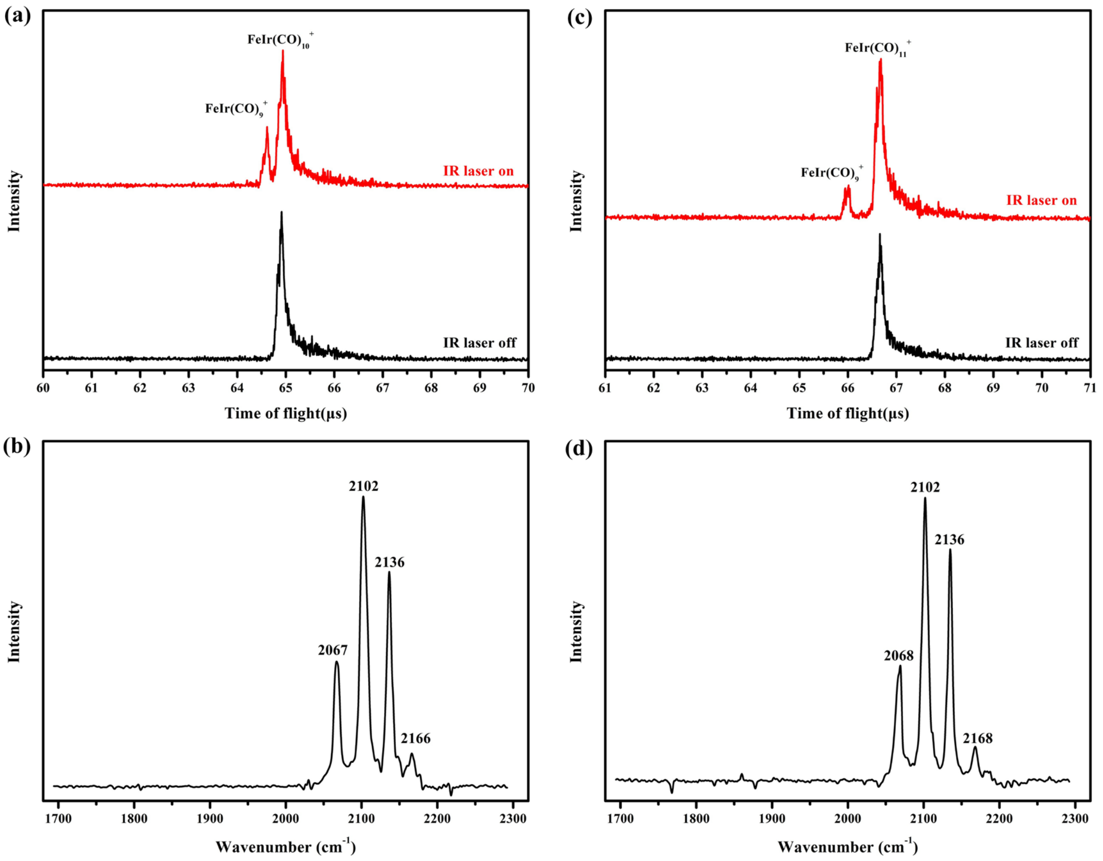

Figure S3. The laser-induced photofragmentation mass spectrum of mass-selected $FeIr(CO)_{10}^+$ (a) and the resulting infrared photodissociation spectrum (b); the laser-induced photofragmentation mass spectrum of mass-selected $FeIrCO)_{11}^+$ (c) and the resulting infrared photodissociation spectrum (d).

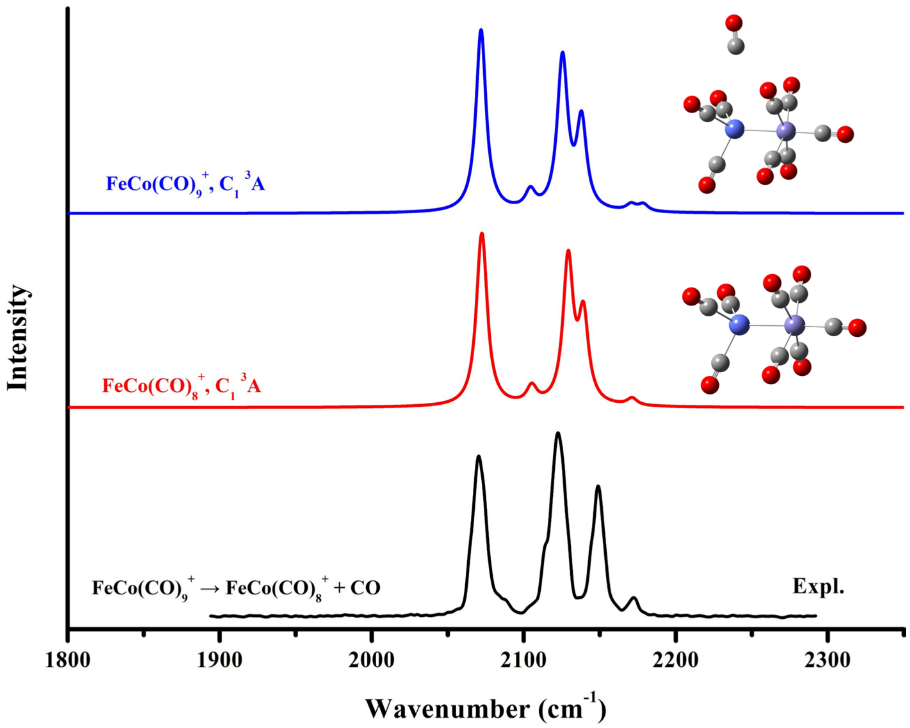

Figure S4. The experimental infrared photodissociation spectrum of $FeCo(CO)_9^+$ and the simulated vibrational spectra of the saturated $FeCo(CO)_8^+$ and solvated $FeCo(CO)_9^+$ cation complexes in the carbonyl stretching frequency region.

Table S1. The calculated charge distribution of $FeM(CO)_9^+$ based on natural population analysis.

Complex	Site	NPA charge	Spin population
$FeRh(CO)_9^+$	Fe1	-2.221	-
(C_{4v} $^1A_1$)	Rh2	-1.068	-
	C7-O8	0.572	-
	C9-O10	0.519	-
	C3-O4	0.412	-
$FeIr(CO)_9^+$	Fe1	-2.228	-
(C_{4v} $^1A_1$)	Ir2	-0.837	-
	C7-O8	0.581	-
	C9-O10	0.531	-
	C3-O4	0.340	-
$FeCo(CO)_8^+$	Fe1	-2.341	0.020
(C_1 ^3A)	Co2	-0.300	1.816
	C7-O8	0.573	-0.006
	C9-O10	0.539	0.002
	C3-O4	0.305	0.055

Table S2. Calculated thermodynamic energy (electronic energy including zero-point vibration energy correction) of free CO, triplet $FeCo(CO)_8^+$, and singlet $FeCo(CO)_9^+$, as well as the relative energy of singlet $FeCo(CO)_9^+$ to $FeCo(CO)_8^+$ plus CO at the B3LYP, TPSSh, TPSS, and PBE level with def2-TZVPP basis set, respectively.

	$FeCo(CO)_8^+$ (a.u.)	CO (a.u.)	$FeCo(CO)_9^+$ (a.u.)	ΔE (eV)
B3LYP	-3553.428911	-113.340223	-3666.786101	-0.462
TPSSh	-3553.450647	-113.354550	-3666.833857	-0.780
TPSS	-3553.682697	-113.369579	-3667.086705	-0.937
PBE	-3552.033234	-113.229441	-3665.308458	-1.246

Table S3. The cartesian coordinates (Å) of the structures of $FeM(CO)_9^+$ optimized at B3LYP/def2-TZVPP level.

Complex	Atom	x	y	z
$FeRh(CO)_9^+$	Fe	0	0	-1.64778
(C_{4v}, $^1A_1$)	Rh	0	0	1.519343
	C	1.372805	1.372805	1.794769
	O	2.158761	2.158761	1.978374
	C	-1.37281	-1.37281	1.794769
	O	-2.15876	-2.15876	1.978374
	C	0	0	-3.49269
	O	0	0	-4.62126
	C	0	1.827068	-1.39055
	O	0	2.953495	-1.26911
	C	1.372805	-1.37281	1.794769
	O	2.158761	-2.15876	1.978374
	C	1.827068	0	-1.39055
	O	2.953495	0	-1.26911
	C	0	-1.82707	-1.39055
	O	0	-2.9535	-1.26911
	C	-1.37281	1.372805	1.794769
	O	-2.15876	2.158761	1.978374
	C	-1.82707	0	-1.39055
	O	-2.9535	0	-1.26911
$FeIr(CO)_9^+$	Fe	0	0	-1.80691
(C_{4v}, $^1A_1$)	Ir	0	0	1.266732
	C	1.369596	1.369596	1.564447
	O	2.153904	2.153904	1.779444
	C	-1.3696	-1.3696	1.564447
	O	-2.1539	-2.1539	1.779444
	C	0	0	-3.65234
	O	0	0	-4.78032
	C	0	1.835664	-1.57353
	O	0	2.962645	-1.47269
	C	1.369596	-1.3696	1.564447
	O	2.153904	-2.1539	1.779444
	C	1.835664	0	-1.57353
	O	2.962645	0	-1.47269
	C	0	-1.83566	-1.57353
	O	0	-2.96265	-1.47269
	C	-1.3696	1.369596	1.564447
	O	-2.1539	2.153904	1.779444
	C	-1.83566	0	-1.57353
	O	-2.96265	0	-1.47269
